# Supplementary material for: From sequence to enzyme mechanism using multi-label machine learning
Source: BMC Bioinformatics. 2014 May 19;15:150. doi: 10.1186/1471-2105-15-150 (PMC4229970; doi:10.1186/1471-2105-15-150)
Supplement: Additional file 2 — Java code of ml2db. Additional file ml2db_code.tar.gz contains the Java source code to run the multi-label machine learning experiments and save the results to database. The code’s Javadoc is included. [file 1471-2105-15-150-S2.zip › additional file 2/ml2db/ml2db/doc/main/ml2db/mulan/learn/evaluation/EvaluationParameters.html]

EvaluationParameters


JavaScript is disabled on your browser.


- Overview
- Package
- Class
- Use
- Tree
- Deprecated
- Index
- Help

- Prev Class
- Next Class

- Frames
- No Frames

- All Classes

- Summary:
- Nested |
- Field |
- Constr |
- Method

- Detail:
- Field |
- Constr |
- Method


main.ml2db.mulan.learn.evaluation

## Class EvaluationParameters

- java.lang.Object
- - main.ml2db.mulan.learn.evaluation.EvaluationParameters

- ---

    

  ```
  public class EvaluationParameters
  extends java.lang.Object
  ```

  Contains the evaluation parameters (micro-macro precision, micro-macro
  accuracy etc.) of a Multi label Mulan experiment.

  Version:
  :   8 Jun 2010

  Author:
  :   Luna De Ferrari ldf(at)st-andrews.ac.uk

- - ### Constructor Summary

    Constructors

    Constructor and Description

    `EvaluationParameters()`
  - ### Method Summary

    Methods

    | Modifier and Type | Method and Description |
    | --- | --- |
    | `static java.util.TreeSet<java.lang.String>` | `getMeasureNames()` Get a list containing the evaluation measure names. |

    - ### Methods inherited from class java.lang.Object

      `equals, getClass, hashCode, notify, notifyAll, toString, wait, wait, wait`

- - ### Constructor Detail


    - #### EvaluationParameters

      ```
      public EvaluationParameters()
      ```
  - ### Method Detail


    - #### getMeasureNames

      ```
      public static java.util.TreeSet<java.lang.String> getMeasureNames()
      ```

      Get a list containing the evaluation measure names.

      Returns:
      :   a list containing the evaluation measure names.


- Overview
- Package
- Class
- Use
- Tree
- Deprecated
- Index
- Help

- Prev Class
- Next Class

- Frames
- No Frames

- All Classes

- Summary:
- Nested |
- Field |
- Constr |
- Method

- Detail:
- Field |
- Constr |
- Method
